# Prevalence and Characterization of Extended-Spectrum β-Lactamase-Producing Antibiotic-Resistant *Escherichia coli* and *Klebsiella pneumoniae* in Ready-to-Eat Street Foods

**DOI:** 10.3390/antibiotics10070850

**Published:** 2021-07-13

**Authors:** Shobha Giri, Vaishnavi Kudva, Kalidas Shetty, Veena Shetty

**Affiliations:** 1 Department of Microbiology, KS Hegde Medical Academy, Nitte (Deemed to be University), Mangalore 575018, India; shobhasmc@yahoo.com (S.G.); pillukudva@gmail.com (V.K.); 2 Department of Plant Sciences, North Dakota State University, Fargo, ND 58102, USA; kalidas.shetty@ndsu.edu

**Keywords:** food safety, RTE street foods, antibiotic resistance, *Escherichia coli*, *Klebsiella pneumoniae*

## Abstract

As the global urban populations increase with rapid migration from rural areas, ready-to-eat (RTE) street foods are posing food safety challenges where street foods are prepared with less structured food safety guidelines in small and roadside outlets. The increased presence of extended-spectrum-β-lactamase (ESBL) producing bacteria in street foods is a significant risk for human health because of its epidemiological significance. *Escherichia coli* and *Klebsiella pneumoniae* have become important and dangerous foodborne pathogens globally for their relevance to antibiotic resistance. The present study was undertaken to evaluate the potential burden of antibiotic-resistant *E. coli* and *K. pneumoniae* contaminating RTE street foods and to assess the microbiological quality of foods in a typical emerging and growing urban suburb of India where RTE street foods are rapidly establishing with public health implications. A total of 100 RTE food samples were collected of which, 22.88% were *E. coli* and 27.12% *K. pneumoniae.* The prevalence of ESBL-producing *E. coli* and *K. pneumoniae* was 25.42%, isolated mostly from chutneys, salads, paani puri, and chicken. Antimicrobial resistance was observed towards cefepime (72.9%), imipenem (55.9%), cefotaxime (52.5%), and meropenem (16.9%) with 86.44% of the isolates with MAR index above 0.22. Among β-lactamase encoding genes, *bla*_TEM_ (40.68%) was the most prevalent followed by *bla*_CTX_ (32.20%) and *bla*_SHV_ (10.17%). *bla*_NDM_ gene was detected in 20.34% of the isolates. This study indicated that contaminated RTE street foods present health risks to consumers and there is a high potential of transferring multi-drug-resistant bacteria from foods to humans and from person to person as pathogens or as commensal residents of the human gut leading to challenges for subsequent therapeutic treatments.

## 1. Introduction

Ready-to-eat (RTE) street foods have food safety challenges globally and are defined as foods for immediate consumption or subsequent use without further processing or preparation and are sold as common street foods in small roadside outlets [1]. RTE street foods could be consumed raw or cooked, hot or cold. Fruits (cut fruits or fruit mixtures) bought directly from street vendors, or local street markets could also be considered RTE if eaten immediately, i.e., without necessarily having to wash, peel, or cut before consumption [2]. As the world urbanizes more rapidly, there is a higher dependency on street foods and specifically RTE foods among the urban population due to time and cost pressures [3]. The consumption of street food increases the potential risk of foodborne illnesses such as diarrhea or traveler’s diarrhea [4]. The entry points of foodborne pathogens are from readily contaminated foods from different sources which lack structured hygiene practices during processing, preparation, and storage [5,6].

The World Health Organization (WHO) estimates that foodborne and waterborne diarrheal diseases kill around 2.2 million people annually. According to the Centre for Disease Control and Prevention (CDC), millions of illnesses occur throughout the world each year due to foodborne pathogens [7]. Further, with the increased use of antibiotics to treat foodborne bacterial illness it increases antimicrobial resistance and subsequent ineffectiveness of antibiotics [8]. Therefore, this has resulted in increased infections caused by these Multidrug-resistant (MDR) organisms and is a serious problem encountered throughout the world as they are associated with reduced therapeutic options, increased mortality, and a lengthy hospital stay [9,10]. *Escherichia coli*, for example, have become a dangerous foodborne pathogen globally responsible for gastroenteritis epidemics in North America, Europe, Asia, and Africa and many strains have been frequently implicated in undercooked foods, contaminated ground beef, raw milk, unpasteurized cider and apple juice, bean sprouts, or fresh leafy vegetables such as lettuce and spinach [11] and have a potential for the emergence of antibiotic resistance. Similarly, resistance in *K. pneumoniae* has been observed in various community sources including raw vegetables and RTE foods over the years and there are published reports of *K. pneumoniae* having developed an acquired resistance to carbapenems, the last-line drugs [12].

Among challenges of antibiotic resistance, the increased presence of ESBL-producing foodborne bacteria in street foods is a significant risk for human health because of its epidemiological importance [13]. Hence, there is a need to screen these ready-to-eat street foods to reduce their dissemination into the environment, including food and water. The present study was undertaken to evaluate the potential burden of ESBL-producing antibiotic-resistant *Escherichia coli* and *Klebsiella pneumoniae* contaminating the RTE street foods and to assess the microbiological quality of foods in a typical emerging and growing urban suburb of India where RTE street foods are being rapidly establishing with public health implications.

## 2. Materials and Methods

### 2.1. Sample Collection

A total of 100 RTE street food samples were purchased and collected between June and October 2019 (location: Deralakatte suburb of Mangalore city in India) to analyze for ESBL-producing antibiotic-resistant *E. coli* and *K. pneumoniae.* The samples consisted of vegetarian and non-vegetarian salads, different sandwiches, a variety of chutneys, sauces and dressings, instant noodles and pasta, vegetarian and non-vegetarian fritters, paani puri water and potato mix (a local Indian street food), mayonnaise, samosas and cutlets, chaats like bhelpuri (mixture of puffed rice with sweet and sour chutney) and potato chaat (mixture of potatoes with green and red chutney, curd, cut vegetables and some dry masala powder), patties with the filling of eggs/ vegetables /chicken, chicken sausages and salami, wet and dry pickles, fruits and vegetable juices, cakes and muffins, and different types of cheese. The food samples mentioned above were collected in a sterile plastic container from a range of street food options such as the local food stalls, canteens, roadside vendors, and small outlets and transported to the laboratory at 4 °C.

### 2.2. Isolation and Identification of Organisms

Approximately 25 g from each sample was mixed and macerated with 3 mL of brain heart infusion broth (BHI) (HiMedia, Mumbai, India) in a sterile mortar and pestle. Then, the samples were homogenized by mixing into 225 mL of buffered peptone water (HiMedia, Mumbai, India) followed by incubation for 24 h at 37 °C. The enrichment was streaked onto MacConkey agar (MA) and Nutrient agar (NA) (HiMedia, Mumbai, India) [14]. Plates were incubated at 37 °C for 24–48 h. Up to three suspected *E. coli* and *K. pneumoniae* colonies from each plate were subcultured onto MA with cefotaxime (1 μg/mL) for the detection of potential ESBL producers followed by incubation at 37 °C for 24 h. Bacterial isolates were subjected to a diverse array of standard biochemical tests to identify *E. coli* and *K. pneumoniae* [15].

### 2.3. Antimicrobial Susceptibility Testing

The antibiotic susceptibility testing was done and interpreted using standard Kirby-Bauer disk diffusion technique on Muller-Hinton agar (MHA) (HiMedia, Mumbai, India) using a panel of 23 different commercially available antibiotic disks (HiMedia, Mumbai, India) which included: ampicillin (AMP, 30 μg), amikacin (AK, 30 μg), amoxyclav (AMC, 20/10 μg), ceftazidime (CAZ, 30 μg), ceftazidime clavulanic acid (CACL, 30/10 μg), ceftriaxone (CTR, 30 μg), cefotaxime (CTX, 30 μg), cefuroxime (CFU, 30 μg), cefepime (CPM, 30 μg), cefoperazone sulbactam (CFS, 75/15 μg), co-trimoxazole (COT, 25 μg), chloramphenicol (C, 30 μg), ciprofloxacin (CIP, 5 μg), ertapenem (ETP, 10 μg), erythromycin (E, 15 μg), gentamicin (GEN, 10 μg), imipenem (IMP, 10 μg), meropenem (MRP, 10 μg), nalidixic acid (NA, 30 μg), nitrofurantoin (NIT, 300 μg) piperacillin-tazobactam (PT, 100/10 μg), tetracycline (TET, 30 μg), and tigecycline (TG, 15 μg). The inhibition zones were measured and interpreted as sensitive or resistant as recommended by the Clinical and Laboratory Standards Institute (CLSI) [16] guidelines using ATCC (USA) standard culture of *Escherichia coli* 25922 and *Klebsiella pneumoniae* 76003 as controls. In case of tigecycline, susceptibility was determined according to USA-FDA breakpoints [17]. The multiple antibiotic resistance (MAR) index was determined as the ratio of the total antibiotics used to the number of antibiotics to which the bacterial isolate was resistant [18].

### 2.4. Phenotypic Detection of ESBL

The detection of ESBL was done using double disk synergy test (DDST) and combined disc diffusion test (CDDT) [16]. For DDST, the isolates were swabbed onto MHA and tested for antibiotic resistance to amoxyclav (20 µg/10 µg), ceftazidime (30 µg/mL), and cefotaxime (30 µg/mL). Upon incubation at 37 °C for 18–24 h, ESBL production was detected by the formation of zone of inhibition around the cephalosporins that increases towards the amoxyclav resulting in synergy formation. For CDDT, isolates were lawn cultured on MHA and tested against ceftazidime (30 µg) and cefotaxime (30 µg) with and without clavulanic acid. Plates were incubated at 37 °C for 24 h. A zone difference of ≥5 mm between the disk of cephalosporin and cephalosporin/clavulanate combination, confirmed for ESBL production. The isolates were also confirmed using triple ESBL detection strip Ezy MIC ^TM^ (MIX^+^/MIX) (HiMedia, Mumbai, India) as per the manufacturer’s instructions.

### 2.5. Detection for Metallo-β-Lactamases (MBL) and Minimum Inhibitory Concentration (MIC)

Screening for metallo-β-lactamase was tested for isolates showing resistance to any of the carbapenems (imipenem/meropenem) by DDST [19] and confirmed by using meropenem (MRP) and MRP with ethylene-diamine-tetra acetic acid (EDTA) Ezy MIC^TM^ Strip (HiMedia, Mumbai, India) method as per the manufacturer’s instructions. *K. pneumoniae* ATCC 2146 strain was used as control. The results were interpreted as MBL positive when the ratio of the value obtained for MRP to the value of MRP + EDTA was >8 or if the zone was observed on the side coated with MRP + EDTA and no zone observed on the opposite side coated with meropenem.

The MIC against meropenem for all MBL positive isolates was determined using Ezy MIC^TM^ strips (HiMedia, Mumbai, India) as per the manufacturer’s instructions and the results were read as per CLSI breakpoints [16]. Bacterial cultures were grown for 6–7 h in 5 mL of Mueller–Hinton Broth (MHB) (HiMedia, India), after which they were lawn cultured onto MHA plates. The E-strips were placed on the center of the dried plates aseptically using an applicator followed by incubation at 37 °C for 24 h. The MIC of the isolate was read as the value where the ellipse intersects the MIC scale on the strip.

### 2.6. DNA Extraction

Bacterial DNA was extracted using the Cetyl trimethyl ammonium bromide (CTAB) method [20]. The bacterial culture grown overnight at 37 °C in LB broth was centrifuged at 11,200× *g* for 5 min. The bacterial pellet obtained was resuspended in 567 μL of 1× TE buffer (10 mM Tris-HCL, 1 mM EDTA; pH: 8.0), 30 μL of 10% SDS and 3 μL of 20 mg mL^−1^ proteinase K. The mixture was incubated at 37 °C for 1 h following which, 100 μL of 5 M NaCl and 80 μL of CTAB/ NaCl solution was added, mixed thoroughly, and incubated for 10 min at 65 °C in a water bath. An equal volume of chloroform/ isoamyl alcohol (24:1) mixture was added, vortexed, and centrifuged at 11,200× *g* for 5 min. The aqueous phase was transferred to a fresh tube and extracted by adding an equal volume of phenol/chloroform/isoamyl alcohol (25:24:1) mixture and by centrifuging at 11,200× *g* for 10 min. The supernatant was transferred to a fresh tube, and DNA was precipitated by adding 0.6 volumes of isopropanol followed by centrifugation at 11,200× *g* for 5 min. DNA pellet was washed with 1 mL of 70% alcohol by centrifugation at 11,200× *g* for 5 min and was vacuum dried. Finally, the DNA was dissolved in 100 μL of 1× TE buffer and stored at −20 °C for further use. The purity and DNA concentration was estimated using a Nanodrop spectrophotometer (ND-1000, V3.3.0, Thermo Fisher Scientific, Waltham, MA, USA).

### 2.7. Detection of Genes Encoding β-Lactamases

Polymerase chain reaction (PCR) assay was carried out to detect three β-lactamase genes *bla*_CTX_, *bla*_SHV_, and *bla*_TEM_ and metallo-β-lactamase gene *bla*_NDM_ in a 30 μL reaction mixture containing 22.2 μL of sterile distilled water, 0.2 μL Taq DNA polymerase, 3 μL of Taq buffer with MgCl2 (TaKaRa, Bangalore, India), 0.6 μL dNTP, 1 μL of 10 pM primer (each forward and reverse) and 2 μL of template DNA. The primer sequences were procured from Juniper Life Sciences, Bangalore, India and the details about their cycling conditions and the references are given in (Table 1). The amplification was carried out in an Eppendorf Mastercycler nexus GX2 (Thermo Fisher Scientific, Waltham, MA, USA) with the optimized PCR program. The amplified products were resolved in 1.5% agarose gel using a 100 base pair DNA ladder (Invitrogen, Thermo Fisher Scientific, Waltham, MA, USA) as a size marker. The gels were visualized in a gel documentation system (Bio-Rad, Hercules, CA, USA).

### 2.8. Statistical Analysis

The data were analyzed using Statistical package for social sciences software version 16 (SPSS Inc., Chicago, IL, USA). Descriptive statistics were performed. Antibiotic resistance data were treated as a binary variable (1= susceptible; 2 = resistant). The collected information was summarized using frequency and percentage for qualitative data. Since the sample size was small and the expected frequencies were more than 20% for a two-by-two table, Fisher’s exact test was used to compare the difference in antibiotic sensitivity for *E.coli* and *K. pneumoniae*. A significant difference was observed if the *p*-value was <0.05.

## 3. Results

### 3.1. Prevalence of Escherichia coli and Klebsiella pneumoniae

In total, 100 samples were collected from hygienic locations such as college cafeterias, coffee shops, and small eatery called tiffin rooms with some level of food safety practices and unhygienic places like street food, local stalls/street shops, and smaller eating locations with fewer food hygiene practices. The samples were divided into eight categories, whose details are given in Table 2. Out of 100 food samples, 118 gram-negative organisms were isolated where 27 (22.88%) isolates were confirmed as *Escherichia coli* and 32 (27.12%) as *Klebsiella pneumoniae.* Among the 27 *E. coli* isolates, 5 (18.51%) of them were ESBL positive and out of 32 *K. pneumoniae* isolates, 11 (34.37%) were ESBL producers. The ESBL positive isolates mainly were from categories 1, 4, 5, and 7: different sauces and chutneys, paani puri/chaats, salads and sprouts, and chicken items, respectively.

### 3.2. Antibiotic Resistance Profile

**Figure 1:**
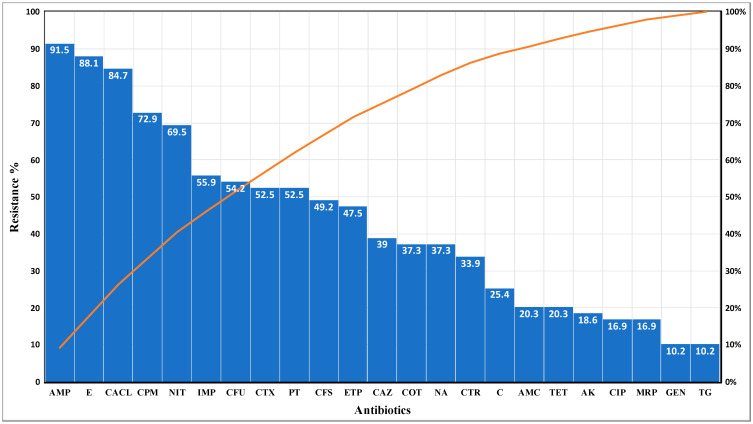
Graph showing the resistance pattern for all antibiotics used in the study.

The isolates showed high resistance to ampicillin (91.5%) followed by erythromycin (88.1%), cefepime (72.9%), imipenem (55.9%), cefotaxime (52.5%), cefoperazone sulbactam (49.2%), and ertapenem (47.5%). The least resistance was to amikacin (18.6%), ciprofloxacin (16.9%), gentamicin (10.2%) and tigecycline (10.2%). The graphical details of the resistance pattern are shown in Figure 1.

Among *E. coli* isolates, highest resistance was to imipenem (96.3%), ampicillin (81.5%), ertapenem (74.1%), cefepime (66.7%), and cefoperazone sulbactum (66.7%) and least resistance to gentamicin (11.1%), amikacin (14.8%) and ciprofloxacin (25.9%). On the other hand, *K. pneumoniae* isolates were 100% resistant to ampicillin, 78.1% resistant to cefepime, 65.6% to cefuroxime, and 62.5% to cefotaxime. The least resistance of *K. pneumoniae* was noted to meropenem (6.3%), ciprofloxacin (9.4%) and gentamicin (9.4%). The resistance percentage for *E. coli* and *K. pneumoniae* are given in Table 3. A significant association (*p* < 0.05) existed between *E. coli* and *K. pneumoniae* isolates with regard to resistance against ampicillin, ampicillin clavulanic acid, ertapenem, imipenem, meropenem, cefoperazone sulbactum, nitrofurantoin, erythromycin, and nalidixic acid (Table 3).

### 3.3. Multidrug Resistance in Escherichia coli and Klebsiella pneumoniae Isolates

The multiple antibiotic resistance (MAR) index of 86.44% of the isolates was above 0.22 which revealed that most isolates were multi-drug resistant, and no isolate was found to be susceptible to all 23 antibiotics tested (Table 4).

### 3.4. Detection of MBL Producers and MIC Value by E-Test

All isolates showing resistance to one or more carbapenem drugs were tested for phenotypic detection of MBL. A total of 12 isolates were found to be positive for MBL by MRP-EDTA Ezy MIC^TM^ Strip test. Out of 12 MBL producing isolates, eight were from *K. pneumoniae* and four were from *E. coli.* Of 12 phenotypically positive MBL isolates, 6 (50%) isolates showed MIC value of >32 μg/mL, 4 (33.3%) isolates showed MIC value ranging from 2–8 μg/mL and 2 (16.7%) from 3–16 μg/mL.

### 3.5. Characterization of β-Lactamase Genes

The molecular detection of β-lactamase genes in this study showed a high prevalence of gene *bla*_TEM_, followed by *bla*_CTX_ and *bla*_SHV_. There were 24 (40.68%) isolates positive for gene *bla*_TEM_, 19 (32.20%) for gene *bla*_CTX_ and 6 (10.17%) isolates were positive for *bla*_SHV_ gene. All three genes were positive in only 2 (3.39%) isolates, and both were *K. pneumoniae.* None of the *E. coli* isolates were positive for all the genes tested. It was noted that 8 (13.56%) isolates showed the presence of both *bla*_CTX_ and *bla*_TEM_, 2 (3.39%) isolates were positive for both *bla*_CTX_ and *bla*_SHV_, and 5 (8.47%) showed the presence of *bla*_SHV_ and *bla*_TEM_ genes together. In 12 (20.34%) of the isolates, *bla*_NDM_ gene was detected (Figures S1–S4).

## 4. Discussion

The present study detected a high prevalence of *Klebsiella pneumoniae* (27.12%) and *Escherichia coli* (22.88%) in 100 different common ready-to-eat street food samples. Our results correlate with other studies for the prevalence of *E. coli*, where 13.43% *E. coli* were isolated from raw salad samples and 43.3% of *K. pneumoniae* isolated from different fresh vegetables [24]. However, there are limited studies from India currently reporting the prevalence of ESBL-producing *K. pneumoniae* from RTE foods and minimal data is available from other countries. The reason seems to be mainly because of different approaches to prevalence estimation. Since there is substantial data available to focus on *E. coli* and other major foodborne pathogens from various sources, this may have resulted in underestimating *K. pneumoniae* as a potential organism prevalent in RTE street foods and ESBL producers. Our study reports that 18.51% of ESBL producers were *E. coli* and 34.37% were *K. pneumoniae.* Contrary to most of the studies screening for ESBL producers, our study reports higher prevalence of ESBL in *K. pneumoniae* [14]. In agreement with our results, a study from South Korea has noted 15.8% of ESBL positive *E. coli* and 84.2% of ESBL-producing *K. pneumoniae* isolated from sprouts [25]. The primary sources of ESBL producers in this study were different chutneys, chaats, paani puri water, vegetarian and chicken salads, and chicken salami and sausages. This can be due to unhygienic practices starting from processing, packaging, storing and handling of food items and a high potential for impact of impaired and contaminated water quality. In addition, the presence of *E. coli* is an indication of fecal contamination of food [26] which also suggests unhygienic practices of the food handlers by using contaminated water for cooking and storing food in contaminated vessels and containers.

The present study reports a high prevalence of multi-drug resistance where 51 isolates fell in the category of MAR index >0.22 out of total 59 *Escherichia coli* and *Klebsiella pneumoniae* isolates. Each isolate was resistant to at least three antibiotics. This suggests an extensive and inappropriate use of antibiotics and chances of contamination from high-risk sources [17] in the food service industry. Many studies from India have reported extensive use of antibiotics in poultry [27] and agriculture [28,29] which provides insights into the prevalence of the high antibiotic-resistant bacterial pathogens from RTE street foods. The resistance towards cefepime (72.9%), imipenem (55.9%), cefotaxime (52.5%), and meropenem (16.9%) observed from the current study should be viewed seriously as these antibiotics are classified under the category of lifesaving drugs used in treating serious infections [30,31].

Resistance to beta-lactams highlights the need for continuous monitoring of antibiotic-resistant patterns of *E. coli* and *K. pneumoniae.* Carbapenems are the choice of drug for ESBL-producing organisms [32], but resistance to them leaves treatment solutions with no evident alternative for treatment. A recent report from the Infectious Diseases Society of America (IDSA) listed ESBL-producing *Escherichia coli* among the most important six drug-resistant bacteria to which new therapies are urgently needed [33]. Therefore, this study suggests appropriate measures to be taken for controlling the dissemination of resistant genes containing pathogens by avoiding indiscriminate use of antibiotics without prescriptions for treatments and improving sanitation and hygiene standards for RTE street foods with food handling procedures and practices for food safety. This study has certain limitations. First, a relatively small sample size, and secondly, a limited geographical area selected for sample collection accounting for it, and hence, the results may not be generalized to other sites. Future studies with a more significant number of samples from a wide selected geographical area will provide more comprehensive information on contamination of RTE street foods by multi-drug-resistant bacteria.

## 5. Conclusions

This study indicates that contaminated RTE street foods with multi-drug-resistant bacteria collected from street food serving cafeterias and local small-scale food vendors present health risks to consumers. Also, there is a high chance that the multi-drug-resistant genes from one bacteria could horizontally transfer to another among the Enterobacteriaceae family with the help of plasmids and thereby transferring antimicrobial resistance leading to difficulties in selecting and using appropriate therapeutic treatments. Hence appropriate measures should be taken for controlling the dissemination of these resistant genes by planning and following proper antibiotic stewardship regimes in the community. In addition, knowledge about proper sanitation, use of clean water for drinking and cooking, cleaning of utensils and containers for storing food items is essential, along with spreading awareness among the local street food vendors and handlers towards hygienic practices. This study’s insights and future extensions also call for harmonizing food safety practices and training for street food establishments on a continuous basis with oversight from local municipalities regulating these food service enterprises.

## Figures and Tables

**Table 1:** Primers used for the amplification of ESBL and metallo-β-lactamase gene.

Primers	Oligonucleotide Sequences (5′-3′)	Thermal Cycling Conditions	Product Size (bp)	Reference
*bla*_CTX-M_	F: ACGTTAAACACCGCCATTCC R: TCGGTGACGATTTTAGCCGC	95 °C 5 min (1 cycle) 94 °C 15 s, 60 °C 1 min, 72 °C 1.3 min (30 cycles), 72 °C 4 min (1 cycle)	356	[21]
*bla*_SHV_	F: ATTTGTCGCTTCTTTACTCGC R: TTTATGGCGTTACCTTTGACC	94 °C 5 min, 94 °C 30 s (30 cycles), 52 °C 30 s, 72° 50 s, 72 °C 10 min	1018	[22]
*bla*_TEM_	F: CTCACCCAGAAACGCTGGTG R: ATCCGCCTCCATCCAGTCTA	95 °C 5 min (1 cycle), 94 °C 15 s, 63 °C 1 min, 72 °C 1.3 min (30 cycles), 72 °C 4 min (1 cycle)	569	[21]
*bla*_NDM_	F: CAACTGGATCAAGCAGGAGA R: TCGATCCCAACGGTGATATT	94 °C 10 min, 94 °C 1 min, 56 °C 30 s (35 cycles), 72 °C 30 s, 72 °C 10 min	291	[23]

**Table 2:** *Escherichia coli* and *Klebsiella pneumoniae* isolates from each category.

Category	Type of Food Items (n = 100)	*E. coli* Isolates (%) (n = 27)	*K. pneumoniae* Isolates (%) (n = 32)
1	Chutney/sauces/dressings/wet and dry pickles (n = 14)	5 (18.51)	7 (21.87)
2	Fried items like samosa, cutlets, different types of veg fritters (n = 12)	2 (7.40)	2 (6.25)
3	Instant cup noodles and pasta, muffins (n =10)	3 (11.11)	1 (3.12)
4	Paani puri and Chaats (n= 15)	5 (18.51)	9 (28.12)
5	Salads and sprouts (n = 14)	4 (14.81)	6 (18.75)
6	Egg items (Eggs puffs, egg fritters, egg sandwiches) (n = 12)	3 (11.11)	2 (6.25)
7	Chicken sausages and Salami (n = 11)	3 (11.11)	4 (12.5)
8	Juices, Cheese and confectionaries (n = 12)	2 (7.40)	1 (3.12)

**Table 3:** Comparison in the difference between antibiotic resistance profiles of *Escherichia coli* and *Klebsiella pneumoniae* isolates using Fisher’s exact test.

Antibiotics	*Escherichia coli* (n = 27)		*Klebsiella pneumoniae* (n = 32)		Fisher’s Exact Test	*p*-Value
	n	%	n	%		
Ceftazidime	7	25.9	16	50.0	0.068	0.059
Ceftazidime clavulanic acid	20	74.1	30	93.8	0.035	0.066
Amikacin	4	14.8	7	21.9	0.526	0.488
Ampicillin	22	81.5	32	100	0.016	0.016 *
Ampicillin clavulanic acid	10	37.0	2	6.3	0.007	0.003 *
Cefotaxime	11	40.7	20	62.5	0.121	0.095
Ceftriaxone	8	29.6	12	37.5	0.589	0.525
Cefuroxime	11	40.7	21	65.6	0.070	0.06
Cefepime	18	66.7	25	78.1	0.386	0.324
Ciprofloxacin	7	25.9	3	9.4	0.070	0.162
Chloramphenicol	7	25.9	8	25.0	1.000	0.935
Ertapenem	20	74.1	8	25.0	0.001	<0.001 *
Imipenem	26	96.3	7	21.9	0.001	<0.001 *
Meropenem	8	29.6	2	6.3	0.018	0.033 *
Cotrimoxazole	11	40.7	11	34.4	0.788	0.614
Gentamicin	3	11.1	3	9.4	1.000	0.826
Cefoperazone sulbactum	18	66.7	11	34.4	0.019	0.013 *
Nitrofurantoin	13	48.1	28	87.5	0.002	0.001 *
Erythromycin	21	77.8	31	96.9	0.028	0.04 *
Nalidixic acid	15	55.6	7	21.9	0.014	0.008 *
Tigecycline	2	7.4	4	12.5	0.280	0.678
Tetracycline	6	22.2	6	18.8	0.757	0.741
Piperacillin tazobactum	15	55.6	16	50.0	0.795	0.67

(* significant *p*-value < 0.05, indicates a significant difference between the *Escherichia coli* and *Klebsiella pneumoniae* towards resistance against the antibiotics).

**Table 4:** Multiple antibiotic resistance (MAR) indices of *Escherichia coli* and *Klebsiella pneumoniae* isolates.

No. of Antibiotics	MAR Index	No. of Isolates (%)
1	0.04	0
2	0.08	0
3	0.13	4 (6.78)
4	0.17	1 (1.69)
5	0.22	6 (10.17)
6	0.26	7 (11.86)
7	0.30	9 (15.25)
8	0.35	1 (1.69)
9	0.39	6 (10.17)
10	0.43	1 (1.69)
11	0.48	3 (5.08)
12	0.52	1 (1.69)
13	0.56	3 (5.08)
14	0.61	9 (15.25)
15	0.65	6 (10.17)
16	0.69	2 (3.39)
17	0.74	1 (1.69)
18	0.78	2 (3.39)

## Data Availability

The data presented in this study are available on request from the corresponding author.

## Supplementary Materials

The following are available online at https://www.mdpi.com/article/10.3390/antibiotics10070850/s1, Figure S1: PCR assay for detection of β-lactamase encoding gene *bla*_CTX_; Figure S2: PCR assay for detection of β-lactamase encoding gene *bla*_SHV_; Figure S3: PCR assay for detection of β-lactamase encoding gene *bla*_TEM_; Figure S4: PCR assay for detection of metallo-β-lactamase gene *bla*_NDM_.
