# Supplementary material for: Prevalence and Characterization of Extended-Spectrum β-Lactamase-Producing Antibiotic-Resistant Escherichia coli and Klebsiella pneumoniae in Ready-to-Eat Street Foods
Source: Antibiotics (Basel). 2021 Jul 13;10(7):850. doi: 10.3390/antibiotics10070850 (PMC8300707; doi:10.3390/antibiotics10070850)
Supplement: Supplementary file 1 [file antibiotics-10-00850-s001.zip › antibiotics-1265998-supplementary.pdf]

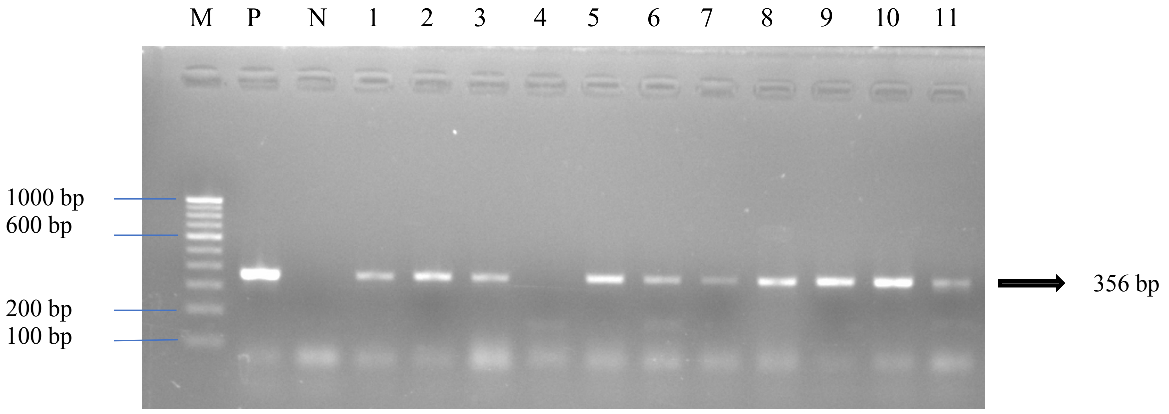

Figure S1: PCR assay for detection of β- lactamase encoding gene bla_{CTX}.

Lane M: Molecular marker 100 bp DNA ladder (Invitrogen, Thermo Fisher Scientific, USA).

Lane P: positive control

Lane N: negative control.

Lanes 1, 2, 3, 5, 6, 7, 8, 9, 10 and 11: positive isolates for gene bla_{CTX} at 356 bp.

Lanes 4: negative isolate for gene bla_{CTX}.

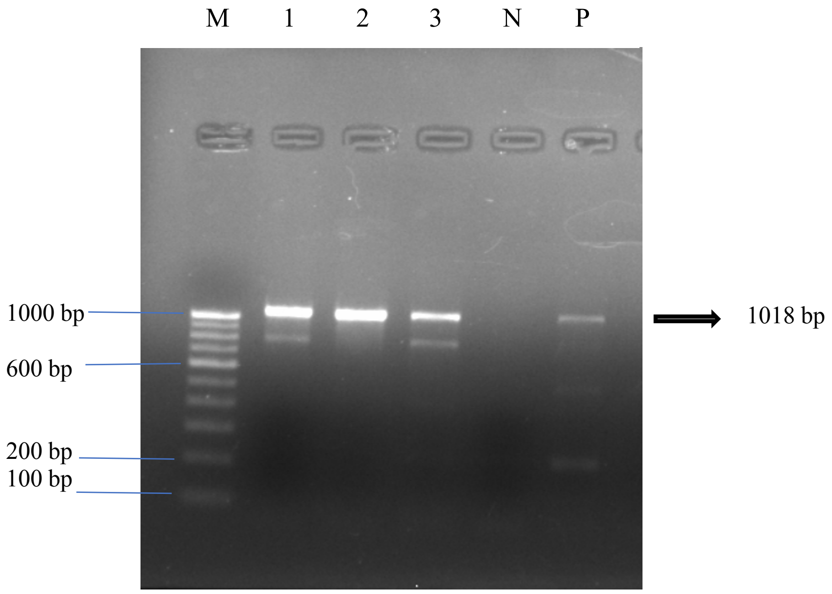

Figure S2: PCR assay for detection of β- lactamase encoding gene *bla*$_{\text{SHV}}$.

Lane M: Molecular marker 100 bp DNA ladder (Invitrogen, Thermo Fisher Scientific, USA).

Lane P: positive control

Lane N: negative control.

Lanes 1, 2 and 3: positive isolates for gene *bla*$_{\text{SHV}}$ at 1018 bp.

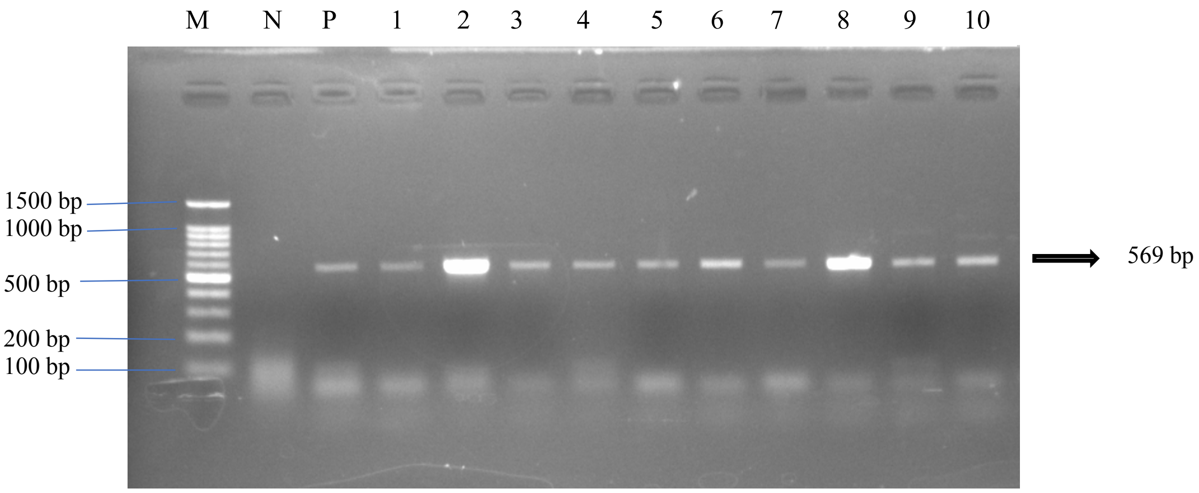

Figure S3: PCR assay for detection of β- lactamase encoding gene *bla*$_{\text{TEM}}$.

Lane M: Molecular marker 100 bp DNA ladder (Invitrogen, Thermo Fisher Scientific, USA).

Lane N: negative control

Lane P: positive control.

Lanes 1 to 10: positive isolates for gene *bla*$_{\text{TEM}}$ at 569 bp.

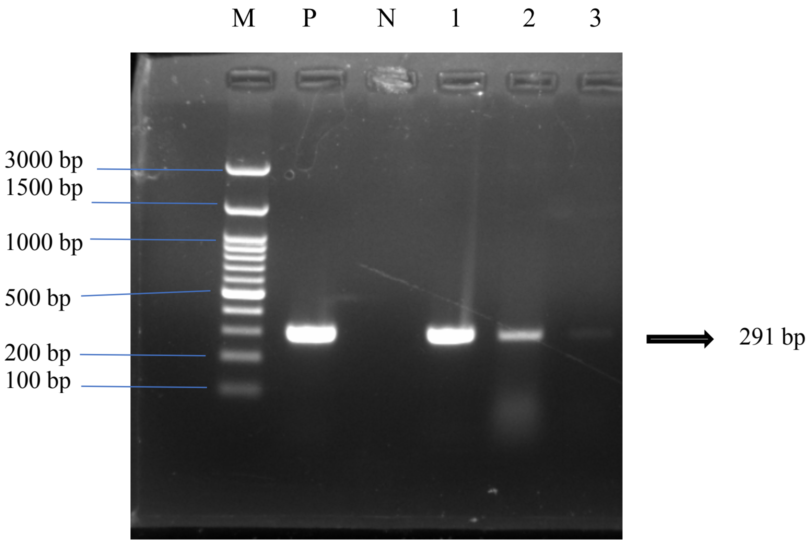

Figure S4: PCR assay for detection of metallo-β- lactamase gene *bla*$_{NDM}$.

Lane M: Molecular marker 100 bp DNA ladder (Invitrogen, Thermo Fisher Scientific, USA).

Lane P: positive control

Lane N: negative control.

Lanes 1, 2 and 3: positive isolates for gene *bla*$_{NDM}$ at 291 bp.
